# Democracy Matters for Child Health

**DOI:** 10.1017/jme.2025.10113

**Published:** 2025

**Authors:** Katherine Hoops, Phillip Cohen, Lee Goeddel, Caroline Fredrickson

**Affiliations:** 1 Anesthesiology and Critical Care Medicine, Johns Hopkins University, Baltimore, United States; 2 Georgetown University Law Center, Washington, United States

**Keywords:** democracy, child mortality, public health, health policy, child health

## Abstract

**Background and Objectives:**

The influence of democracy and democratization on health is difficult to disentangle from a complex web of factors such as population characteristics and social determinants of health. The goal of this study was to begin to characterize the roles of the individual attributes of democracy on a key measure of health, mortality rates among female children under five years of age.

**Methods:**

We conducted a retrospective observational cohort study utilizing data over a study period from 1975–2021 with data from 173 countries. We utilized publicly available data from the Global State of Democracy Indices (GSoD) and the United Nations Inter Agency Group for Child Mortality Estimation (UN-IGME) databases.

**Results:**

Our data support prior work showing that strength of democracy is associated with improved population health measures. Stronger democracies are associated with improvements in female child mortality, even controlling for within-country variation over time and for income level. This relationship is most pronounced when examining the relationship between protections of civil rights and child mortality.

**Conclusions:**

Child mortality increases when democracy declines. With declines in democracy worldwide, it is critical that advocates are concerned with the global democratic experience, especially with policies that compromise fundamental rights.

## Introduction

The influence of democracy and democratization on health is difficult to disentangle from a complex web of other factors such as population characteristics and social determinants of health. A small number of previous studies have shown little or no effect of democracy on health, implicating other factors such as country income as being associated with health outcomes.^1^ However, most studies have shown that democratic governance is associated with improved health outcomes including reduced infant mortality comparing before versus after democratization,^2^ reduced child mortality following protest-led (rather than violence-led) democratic transition,^3^ and increased life expectancy based on level of democracy.^4^ One landmark study used data from the Varieties of Democracy project hosted at the University of Gothenburg: implementing a robust analytic model over a 45-year study period from 1970 to 2015, it found that democratic transition was associated with a 3% improvement in life expectancy after 10 years, largely related to reduced mortality from cardiovascular disease and other non-communicable diseases.^5^ Notably, improvements in standardized mortality from noncommunicable disease were lost when the free and fair elections variable was removed from the statistical model, implying that this variable is key.^6^ However, the contributions of various attributes of liberal democracies on health are, as yet, uncharacterized.

**Figure 1.**
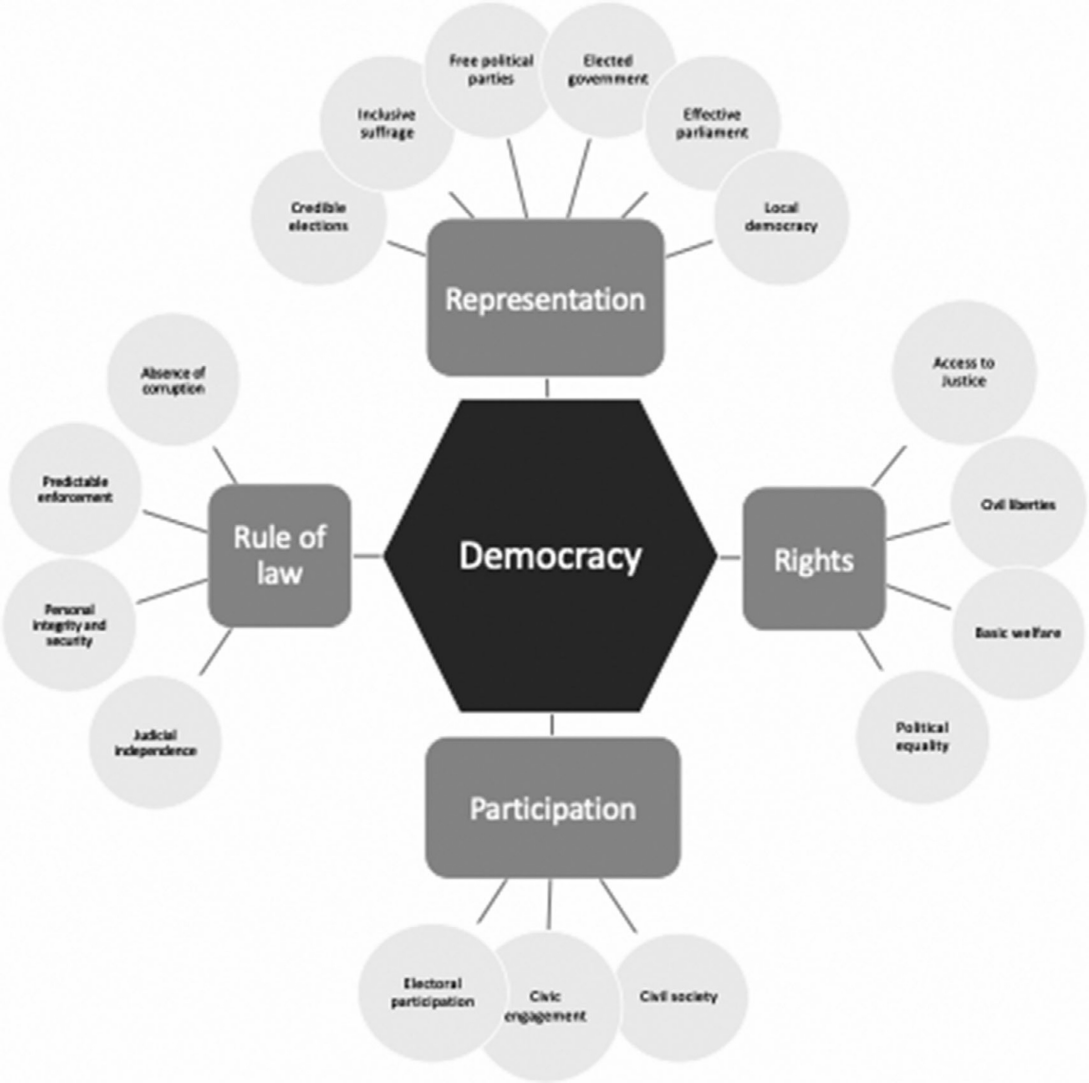
Based on International IDEA conceptual model for the Global State of Democracy Indices depicting attributes, components, and subcomponents.

The International Institute for Democracy and Electoral Assistance (IDEA)^7^ is an intergovernmental organization that undertook the Global State of Democracy (GSoD) Initiative in 2016 in order to contribute publicly-available, evidence-based analyses of the strength and qualities of democracy to better inform policy interventions. Using a theoretical framework that weighs and combines 157 individual indicators collected using multiple data sources (surveys, observational data, and standards from other research groups) into a quantitative dataset that measures performance covering 173 countries. This framework covers a range of attributes of democracy across four domains: representation, rights, rule of law, and participation.^8^ (See Figure 1.)

The goal of this study was to conduct a novel analysis with the best available data to begin to characterize the roles of the individual attributes of democracy on a key measure of health, mortality rates among female children under five years of age (U5MR), hypothesizing that each of the four domains of democracy from the GSoD Index are inversely related to U5MR independent of country income level and time period.

## Methods

Sources of data for this analysis included the GSoD Indices and the United Nations Inter Agency Group for Child Mortality Estimation (UN-IGME) databases. The GSoD Indices database combines data from multiple sources to assess four attributes of democracy (representation, rights, rule of law, and participation) graded on a scale from 0 to 1, with an increasing score corresponding to a higher magnitude of that democratic attribute. In our analysis, we rescaled this to be from 0–100 to easily magnify differences. Separately, the UN-IGME compiles a rigorous estimate of U5MR using vital registration, abridged life tables, survey, and census data.^9^ Critically, after data compilation, adjustments to the preliminary data are made via model processing and in consultation with each country’s Ministry of Health and national statistics office. Overall U5MR is estimated as well as male and female-specific mortality. In this observational, retrospective cohort study, mortality rate among female children under 5 years of age was our primary outcome variable, estimated as the number of deaths per 1,000 live births.

### Statistical analysis

First, Pearson correlation was used to assess the relationship between the four democratic attributes within and across countries and time. Due to correlation between the four democratic attributes, multiple analytic techniques were required to characterize the relationships between each individual democratic attribute and female child mortality. These statistical techniques addressed the multiple dependence level structures within this relationship that could predictably introduce bias in the analysis and obscure the true nature of the relationship including country level differences, regional differences and, perhaps most importantly, changes in democracy and female child mortality over time. Therefore, their relationship and changes over time must also be considered (time dependency in the relationship).

A visual assessment of the relationship between the attributes was performed by plotting all country level data for a given year and regressing a best fit line to visualize the strength and direction of correlation over a series of all included years. Also, to examine the variance between attributes of democracy and under-5 child mortality in the most recent year for which these data were available (2020), box plots were generated within each attribute by the following categories: countries less than the 25^th^ percentile for child mortality, countries between the 25^th^ to 75^th^ percentile for child mortality, and countries greater than the 75^th^ percentile for under-five female child mortality. Finally, change in each attribute was then plotted against change in mortality from 2015 to 2020 to visualize the potential relationships between changes in democracy versus change in mortality.

Then, to test the primary hypothesis of a relationship between the democratic attributes and female U5MR, a primary analysis using mixed-effects multivariable regression was performed. To assess within-country variation over time, country random-effects mixed modeling regression was used. This controls for unique unobserved characteristics in each country and allows for a different underlying effect within each country that together have a quantifiable distribution. Child mortality was regressed on each democratic attribute separately with both country and year random effects. Country income level was identified as a potential confounder of the relationships between democracy and female child mortality; therefore, country income level in 2021, available within the UN-IGME dataset through the World Bank, was adjusted for in all models.

A priori, multiple sensitivity analyses were defined for the primary analysis. Because country income level may have significant effect on female U5MR, the primary analysis on the highest and lowest income countries were deployed separately. Furthermore, because much of the increase in democratic attributes occurred before 1990, in order to understand these relationships in the most recent era, analyses were performed separately for those observations before and after the year 2000. To adjust for multiple comparisons in the primary analysis Bonferroni correction was utilized. All analysis was performed using Stata 17 (College Station, TX).

## Results

All data from the GSoD database from 1975 to 2021 were included; 136 of the 173 countries had complete data for the entire period and 169 countries had complete data from 1985 to 2021. Child mortality data from the UN-IGME database, including male and female U5MR, was available for all countries except East Germany, Kosovo, Czechia, Moldova, North Korea (10 intermittent years missing), Serbia, Suriname, and Taiwan. In total, there were 230 missing estimates of child mortality unable to match to the GSoD database, but 7488 measurements of each democratic attribute were successfully matched to child mortality measurements. Thus, only 3% (230/7718) of data were unavailable for analysis.

**Figure 2.**
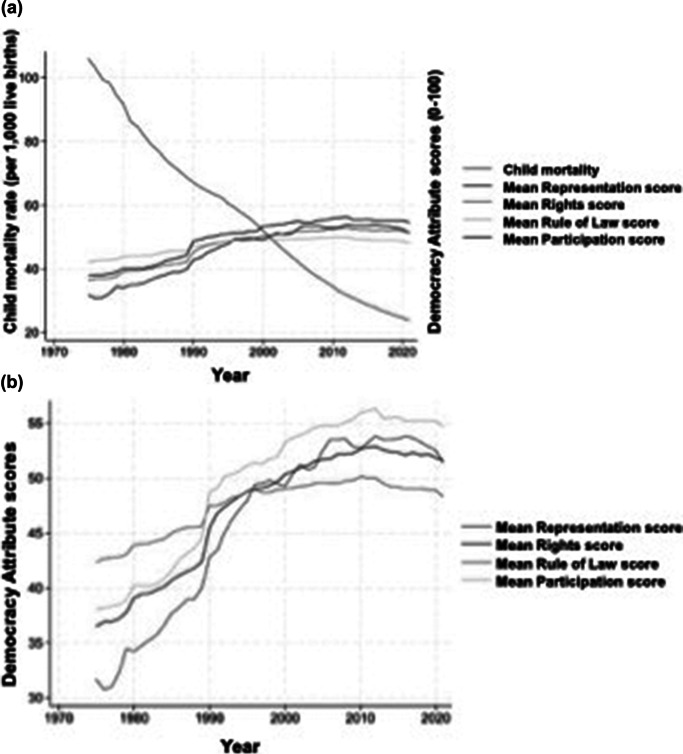
(a) Global trend in child mortality (deaths/1000 live births) and attributes of democracy (scaled to 0 to 100) over time (years). (b) Trends in attributes of democracy (scaled from 0 to 100) over time (years).

Overall global mortality for female children under age five decreased from 1975 to 2021 from a mean (standard deviation) of 113 (87) deaths per 1,000 live births in 1975 to 82 (72) deaths per 1,000 live births in 1985, to 25 (26) deaths per 1,000 live births in 2021 (Figure 2a). All four attributes of democracy also increased (Figure 2b) between 1975 and 2021, but attribute levels in 2021 are similar to levels in 2000.

**Figure 3.**
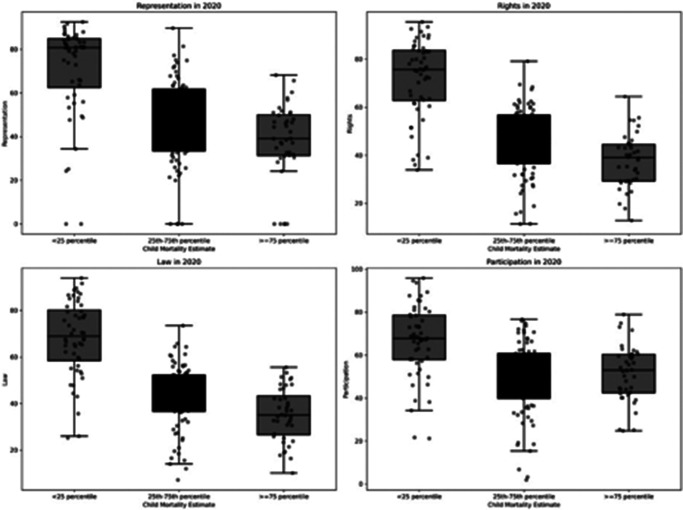
Boxplot Visualization of attributes of democracy grouped by levels of child mortality.

**Figure 4.**
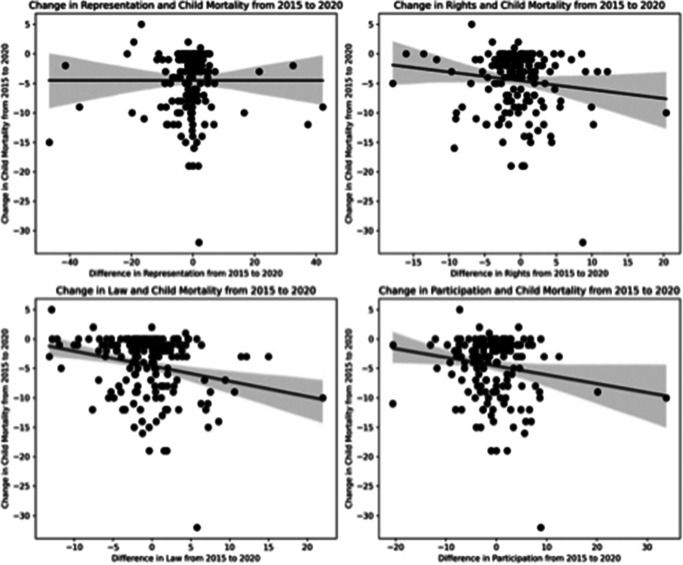
Scatterplot Visualization of trends in changes in attributes of democracy versus changes in female under-five child mortality rates from 2015 to 2020.

In the box plot visualization of data from 2020 (Figure 3), higher levels of each attribute reside in countries within the 25^th^ percentile for child mortality (i.e. the lowest rates of child mortality) and conversely the lowest levels of each attribute reside in countries with estimated child mortality greater than or equal to the 75^th^ percentile (i.e. the highest rates of child mortality). Furthermore, Figure 4 examines changes in democratic attributes in comparison to changes in under-five female child mortality from 2015 to 2020. This suggests that even in the time period from 2015 to 2020, general improvements in democratic attributes were associated with reduced under-five female child mortality.

Pearson correlation coefficients demonstrated partial or substantial correlation between the four attributes of democracy. Rule of law and rights were most correlated (r=.76). In contrast, rule of law and representation were not correlated (r=.02). To avoid collinearity and subsequent error in regression analysis, the relationship between each attribute and female child mortality was evaluated separately. For visualization of possible relationships, each democracy attribute was plotted against female U5MR for each country and a best fit line generated for all countries from years 2014–2021. The main finding in this analysis is that the slope, direction, and magnitude are similar for the relationship across all years within each democratic attribute.

To assess within-country variation over time and adjust for income level, a random effects multilevel regression was used. Table 1 summarizes the results of these four separate models.

**Table 1.** Within-country variation in attributes of democracy over time, adjusted for country income level

Attribute of Democracy	Beta (95% CI)	p-value
Representation	–1.08 (–1.13 – 1.04)	<0.001
Rights	–2.14 (–2.22 – 2.06)	<0.001
Rule of law	–1.11 (–1.21 – 1.0)	<0.001
Participation	–1.69 (–1.75 – 1.62)	<0.001

Each of these models is interpreted where Beta is the change in child mortality (per 1000 live births) independently related to a 1-point increase in each corresponding democratic attribute, where the GSoD indices are rescaled from a 0 to 1 scale to a 0 to 100 scale. For instance, each 1-point increase in the scaled composite score for rights was associated (independent of country income level) with a decrease in female U5MR by 2 per 1000 births. This result reflects analysis within countries across the years of analysis. Additionally, the 95% confidence intervals reflect a precise point estimate with a very low p value which reflects a very low probability that this result is explained by chance. Statistically, a 10-point increase in the scaled composite score for rights over time was independently associated with a decrease in female U5MR by 20 deaths per 1000 live births. Notably, improvements in each democratic attribute were associated with decreases in female U5MR.

### Sensitivity Analyses

To examine the role of income level on the primary analysis, the analysis was repeated using the low-income and high-income subgroup designations in 2021 from the World Bank. These results are displayed in Table 2.

**Table 2.** Sensitivity analysis — Within-country variation in attributes of democracy over time among low- and high-income subgroups

Attribute of Democracy	Low Income		High Income	
	Beta (95% CI)	p-value	Beta (95% CI)	p-value
Representation	–1.92 (–2.1 – –1.75)	<0.001	–0.38 (–0.41 – –0.36)	<0.001
Rights	–4.96 (–5.31 – –4.62)	<0.001	–0.6 (–0.64 – –0.55)	<0.001
Rule of law	–0.51 (–0.95 – –0.62)	0.026	–0.55 (–0.6 – –0.51)	<0.001
Participation	–2.78 (–3.01 – –2.55)	<0.001	–0.49 (–0.53 – –0.45)	<0.001

From 1975 to 2021, changes in democratic attributes had a more significant impact on female child mortality in low-income than high-income countries, within the context of low-income countries having higher female U5MR at the start of the study. However, changes in strength of democratic attributes in both low- and high-income subgroups were associated with changes in child mortality.

Many significant changes in democratic governance occurred in the 1980s to 1990s with resulting global increases in democratic attributes (see Figure 1). Therefore, the primary analysis was also repeated examining the relationships between attributes of democracy and female child mortality in all countries before and after the year 2000 (see Table 3).

**Table 3.** Sensitivity analysis — Within-country variation in attributes of democracy over time in subgroups before and after the year 2000

Attribute of Democracy	1975-1999		2000-2021	
	Beta (95% CI)	p-value	Beta (95% CI)	p-value
Representation	–0.62 (–0.69 – –0.57)	<0.001	–0.21 (–0.27 – –0.15)	<0.001
Rights	–1.3 (–1.39 – –1.22)	<0.001	–0.77 (–0.9 – –0.64)	<0.001
Rule of law	–0.73 (–0.83 – –0.63)	<0.001	–0.26 (–0.38 – –0.14)	<0.001
Participation	–1.04 (–1.1 – –0.97)	<0.001	–0.15 (–0.24 – –0.05)	0.002

Table 3 demonstrates that data prior to 2000 do reflect a higher magnitude of the relationship between changes in the strength of democratic attributes and female child mortality, again within the context of higher starting female U5MR in 1975 than in 2000. However, this sensitivity analysis clearly demonstrates that, in both time periods, each democratic attribute remains strongly and independently associated with child mortality.

## Discussion

The data presented in these analyses support prior work showing that strength of democracy is associated with improved population health measures (Kadamatsu 2012; Reeves and Sochas 2022; Oyèkọ́lá 2023; Bollyky et al. 2019). However, this study adds a novel analysis examining the relationship between specific attributes of democracy — representation, fundamental rights, rule of law, and participation — and female U5MR. While increases in democratization prior to 2000 were associated with greater improvements in child mortality, these relationships have persisted and must be heeded, particularly as the strength of democracy wanes in many countries across the globe (see Figure 2b), and we face a rise of illiberalism or growth of systems that would restrict individual rights and freedoms among even those countries with longstanding traditions of democracy. This analysis holds when controlling for country income level, a major shortcoming of prior analyses, revealing a durable relationship between stronger democratic attributes and reduced female child mortality.

This analysis is the first known to employ the GSoD Indices to examine the role of democracy in child health; these indices add rigor to the study of governance and health as they utilize multiple data sources to generate composite markers of democracy and human rights in organized, discrete components. The isolation of these various attributes of democracy is key. While higher levels of each attribute are associated with lower female U5MR, it is evident that, even controlling for income level and time period, higher scores across domains are most strongly associated with reductions in child mortality over time, underscoring the importance of protection of fundamental rights through access to justice, protection of civil liberties, and social rights and equity. Our data visualizations in Figures 3 and 4 further illustrate this, showing that in very recent years, child mortality is higher in countries with lower levels of democracy but that improvements in democratic attributes were associated with improvements in child mortality.

This study has important limitations. First, there are multiple sources of potential confounding related to both democracy and child mortality that we cannot quantify to include in our model adjustment. Notably, these include some factors that might be proposed as potential confounders that may in fact be complex mediators on the causal pathway between democracy and child mortality. One clear example is the fact that over the course of the study years, there were tremendous advances in pediatric medicine that reduced infant mortality. These advances may have disproportionately benefitted high-income countries like the United States, which also had higher democracy attributes. Whether these medical advances were more likely due to democratic attributes and whether these medical advances were swiftly implemented in democratic nations are inherently more complex questions not answerable by our analysis. However, other interventions also improved infant mortality in lower-income countries. Therefore, this study cannot explain why democracy may be associated with reduced child mortality. Other limitations are related to the data sources themselves. Country income level was not present across time to model as a time-varying covariate; rather, we used the most recent income level data available. Additionally, though they are based on robust data, the democratic attributes and mortality data are, themselves, best estimates of other aggregate data. It is our hope that the evidence of a relationship between democracy and child mortality motivates future study in this topic. Future work will include further refining this exploratory analysis by developing synthetic control models.

## Conclusions

With declines in democracy worldwide, it is critical that advocates, including public health and child health advocates, are concerned with the global democratic experience, especially with policies that infringe on or compromise fundamental rights. Our results underscore the importance of protecting of civil rights through access to justice, protection of civil liberties, and social rights and equity.
